# Utilization of the national cluster of district health information system for health service decision-making at the district, sub-district and community levels in selected districts of the Brong Ahafo region in Ghana

**DOI:** 10.1186/s12913-020-05349-5

**Published:** 2020-06-06

**Authors:** Eliezer Ofori Odei-Lartey, Rebecca Kyerewaa Dwommoh Prah, Edward Apraku Anane, Harry Danwonno, Stephaney Gyaase, Felix Boakye Oppong, Godwin Afenyadu, Kwaku Poku Asante

**Affiliations:** 1 Kintampo Health Research Centre, P. O. Box 200, Kintampo, Ghana; 2 USAID/Ghana Evaluation for Health, Accra, Ghana

**Keywords:** Decision-making, Utilization, Evidence-based, District-level, Community-level, dhis2, Ghana, CHPS

## Abstract

**Background:**

There is growing interest in the use of reliable evidence for health decision-making among low-and middle-income countries. Ghana has deployed DHIMS2 to replace the previously existing manual data harmonization processes.

**Methods:**

This cross-sectional study was conducted in 12 districts comprising 12 district directorates, 10 district hospitals, 29 sub-district health centers, and 38 community health facilities in the Brong-Ahafo Region. Data collection tools were developed based on the Measure Evaluate assessment tools designed for evaluating the performance of routine information systems management tools. Utilization was assessed based on documented evidence and data was analyzed using STATA version 14.

**Results:**

Although 93% of the health facilities studied submitted data unto the DHIMS2 platform, evidence suggested low use of this data in decision-making, particularly at the community level facilities where only 26% of the facilities used data from DHIMS2 to inform annual action plans and even less than 20% examined findings and issued directives for action. At the district level, 58% issued directives based on DHIMS2 information, 50% used DHIMS2 information for Advocacy purposes and 58% gave feedback reports based on DHIMS2 data for action. Functional computers were lacking across all facilities.

**Conclusions:**

Activities relating to the use of DHIMS2 information skew towards data quality checking with less focus on examining findings, making comparisons, and taking action-based decisions from findings and comparisons. Improving factors like internet access, availability of functional ICTs, frequency of supervisory visits, staff training and the provision of training manuals may facilitate the use of DHIMS2 in decision-making at all levels of the district health system.

## Background

In low and middle-income countries (LMICs), routine health information systems (HIS) are generally weak [1], characterized by record duplications, fragmentation, incompleteness, and multiple storage formats [2]. Lately however, LMICs are showing increasing interest in establishing HIS that can generate reliable data for improving health performance [3]. Some LMICs have initiated a sequence of health information system reforms to compile and harmonize data in the country to create a valuable resource for decision-making in the health sector [4, 5]. An example of such major reforms is the adoption of an open-source District Health Information System (DHIS2) by Ghana, Kenya, Tanzania, and Sierra Leone. Such information systems are intended for harmonizing nation-wide data and serve as bases for understanding health patterns, making informed decisions, and forming actions to improve lives in these countries [6–8].

The use of data to support decision-making is an important element of the facility-based information system [9]. Despite this, the availability of high quality and reliable data does not usually result in the utilization of that data. This has consequently resulted in a disjoint between data production, dissemination, and use even though this should not be the case. An evaluation of the HIS reforms in LMICs is important to ensure effective data utilization and evidence-based decision making to guide policies and programs in the health sector [10].

In Ghana, the DHIS2 was rolled out as a national cluster of District Health Information Management System (DHIMS2) to routinely collect and compile health data for decision making. The DHIMS2 platform was implemented by the Ghana Health Service (GHS) in collaboration with the University of Oslo. It is a comprehensive web-based application for remotely compiling data across different levels of a health system into a central storage point. It uses data warehouse principles and a modular structure to allow for customization to distinct needs of different health systems. The introduction of the DHIMS2 platform has led to a reduction in the information transmission bottlenecks/timelines. As of 2013, the DHIMS2 was accessible in 170 out of 216 districts with about 5163 registered users [11]. A recent report from the Ghana Health Service showed that the total number of registered users as at the end of 2016 was over 10,000 [12].

Despite improvements in health information systems in Ghana, there are continuous weaknesses in the health system. This has been attributed to the limited use of evidence for decision-making by health managers and policymakers, particularly at the district, sub-district and community levels. Such practices lead to misallocation of health resources, misplaced prioritization, and unmet healthcare demands. Although a study has been conducted to evaluate the quality of data in the DHIMS2 in Ghana, [13], very little was done to assess how the data was being used for decision–making.

This study aimed at evaluating the utilization of data from DHIMS2 for decision-making at the district, sub-district and community levels in selected districts in the Brong Ahafo Region. In this manuscript, we report on the coverage of DHIMS2 in terms of available modules and variables, the extent to which DHIMS2 information was used to inform the decision-making processes, the perceived capacity of decision-makers to execute certain tasks on the DHIMS2 platform and the factors influencing the effective utilization of DHIMS2 information in decision-making.

## Methods

### Study setting

**Fig. 1:**
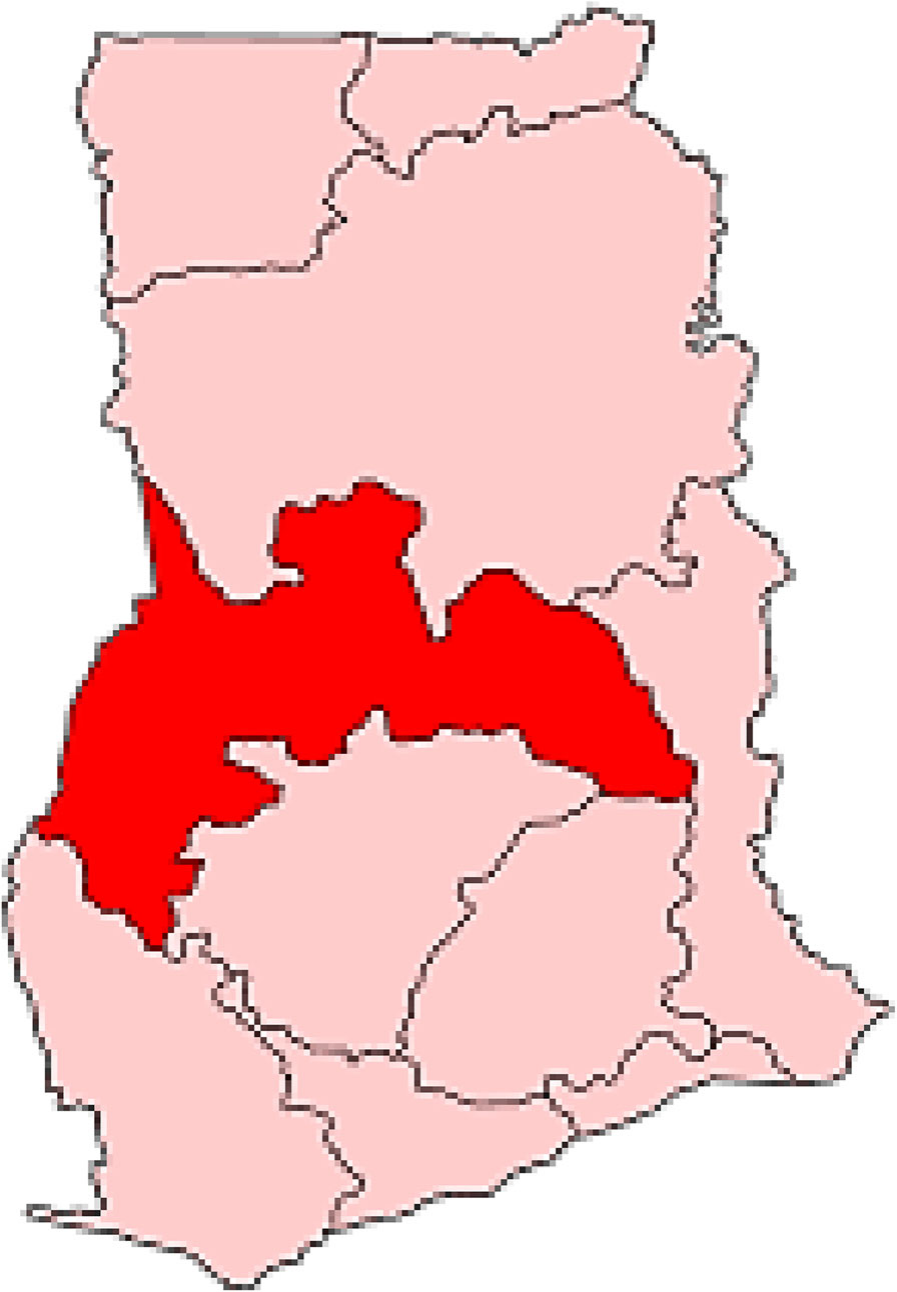
Map of Ghana showing the location of the Brong Ahafo Region. Figure 1 shows the Ghana map. At the time of the study, Ghana had 10 regions as demarcated in the diagram. The Brong Ahafo area where the study was carried out is shaded Red. This file is licensed under the Creative Commons Attribution-Share Alike 3.0 Unported license

The study was conducted in the Brong-Ahafo region of Ghana (Fig. 1). It has 27 administrative districts. Each district is divided functionally into sub-districts. There was one district health directorate in each district, the district health management team (DHMT) responsible for managing health activities throughout the district. For most districts, District Government Hospitals served as a referral hospital to other district facilities. The Regional Hospital was located in Sunyani, the capital of the Brong Ahafo Region.

### Study design

The study was cross-sectional and conducted between January and March 2018 using three approaches. The first approach consisted of a desk review of annual reports, minutes of meetings, periodic performance assessment reports, and feedback reports from the supervisor. The second was a quantitative approach to examine the coverage, knowledge, and use of DHIMS2 data for decision making. This review was undertaken to examine the utilization of the DHIMS2 platform by the health facilities in decision-making. Finally, a qualitative approach was adopted to explore potential enablers and barriers to the effective use of DHIMS2 information in the district, sub-district, and community level decision-making of the health system.

### Study population

**Fig. 2:**
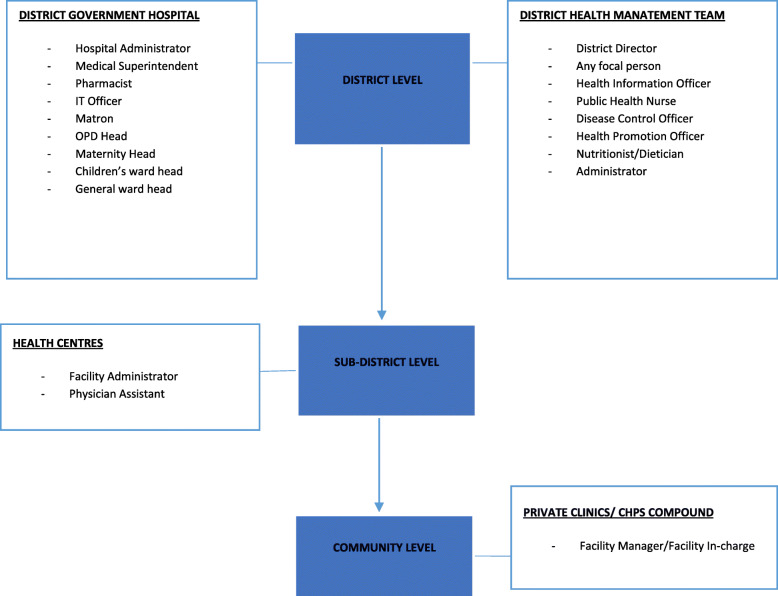
Decision makers identified at the different health system levels. In Figure 2 the shaded blue boxes represent the three stages of the health system as described in the report. At each level, the list of identified decision-makers was classified according to the type of facility

The population of the study was derived from the district health system in the Brong Ahafo Region. The selection of participants was based on the hierarchical structure of the district health system used in DHIMS2. Participants span all three functional levels of district health system facilities, targeting health managers/workers who were involved in decision-making (Fig. 2). At the district level, eight decision-makers were identified from the DHMT and nine from the District Government Hospital (DGH).

In each sub-district, there was one facility referred to as a health center, which played a supervisory role over other health facilities. The health centers were responsible for harmonizing DHIMS2 data from private clinics and community-based health planning and services (CHPS) compounds within their supervisory limits. Two decision-makers were identified at the sub-district level. Health facilities at the community level included private clinics and CHPS compounds. Decision-making at the CHPS compounds was led by a community health officer also referred to as the facility in-charge. At the private clinics, the facility manager led decision making.

### Sampling strategy

**Fig. 3:**
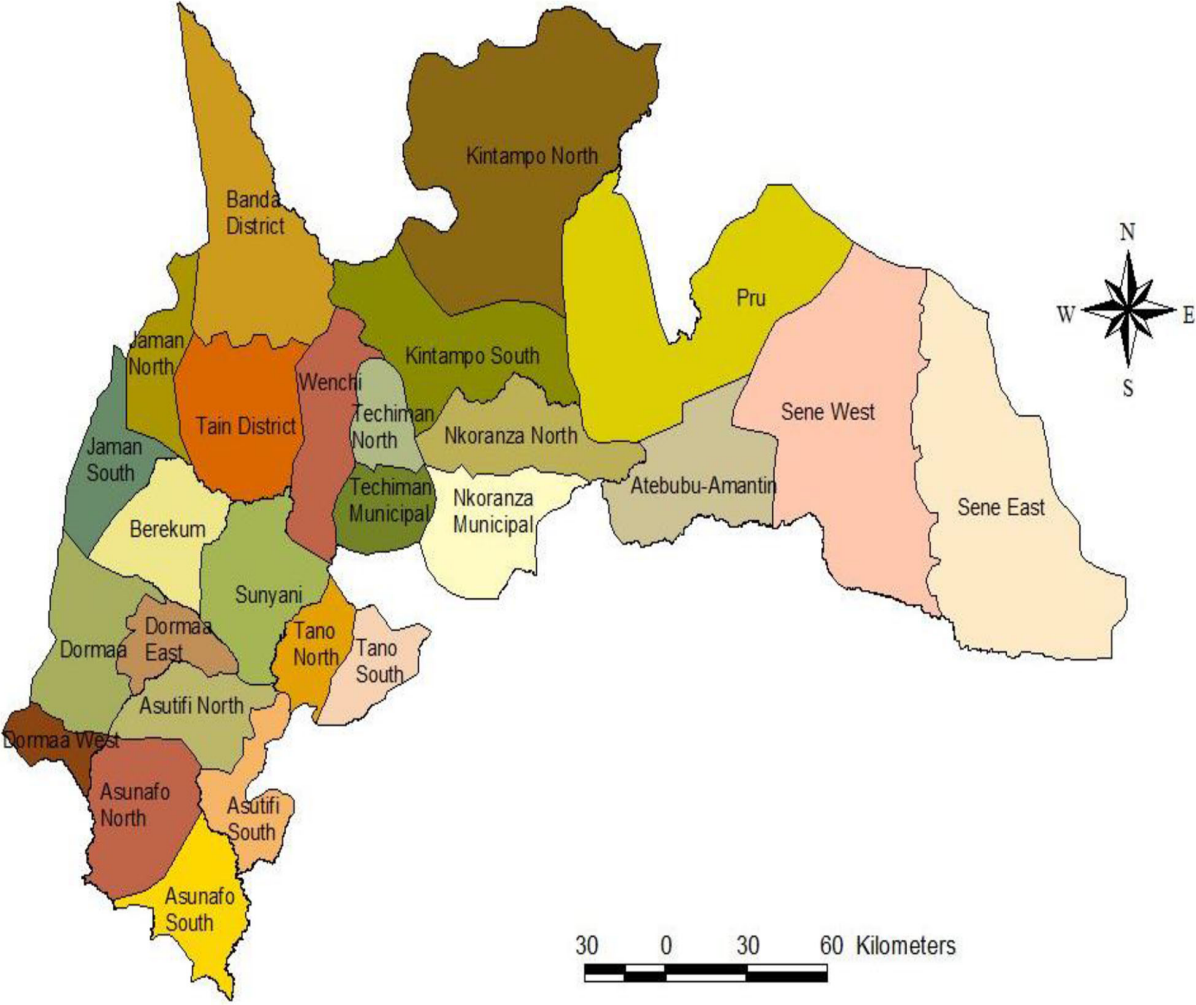
Map of Brong Ahafo Region showing the 27 Districts. The map of the Brong Ahafo area is illustrated in Figure 3. All 27 districts within the area as labeled were shaded by different colors. This file is from the authors’ own source

The selection of the 12 districts was based on the working presence of the Kintampo Health Research Center (KHRC). A total of 12 of the 27 districts (Fig. 3) in the Brong Ahafo region were visited to gather data for the study.

A hierarchical technique was employed in selecting participants for the study. The selection process was non-probabilistic and hierarchical stages corresponded with the district, sub-district, and community levels of the health system. The selection of participants at each level was limited to health personnel in decision-making positions as shown in Fig. 3. The participants were not chosen at random but approached and interviewed based on their willingness to take part in the study.

A total of 23 district-level facilities, including 12 DHMTs and 11 DGHs, were identified. Of the facilities at the district level, 4 participants were selected from each DHMT and 3 participants from each DGH, which added up to a total of 81 participants.

The frame for selecting the sub-district level health facilities was based on those listed in the DHIMS2 database at the time of the study. The sub-district level consisted of a total of 56 health centers, allocated within each district in different proportions. At the sub-district level, a total of 56 health centers were listed. Of the 56 health centers, 29 were acquired by randomly choosing 50% of health centers from each selected district without replacement. Finally, a total of 58 participants were chosen for the study at the sub-district level, two from each health center.

The frame for selecting the community-level health facilities was based on those listed in the DHIMS2 database at the time of the study. A total of 327 health facilities were spread throughout the 12 districts at the community level. Due to time and resource constraints, the selection of facilities at the community level under each of the 29 sub-districts was limited to 2 random facilities without replacement. Of the 327 health facilities, 56 were selected in total and one participant in each was chosen for the study. Table 1 represents the summary statistics of facilities sampled for this study as well as the distribution of selected participants.

**Table 1:** Summary statistics for facility and participant selection in the 12 districts

Health Facilities	Total (in DHIMS2)	Sampled Facilities	Sampled Participants	
			Per facility	Total
District Directorate	12	12	4	48
District/referral Hospital	11	11	3	33
Sub-district	56	29	2	58
Community	327	56	1	56
**Total**	**406**	**107**	**–**	**195**

### Data collection

Before the data collection process, the study team conducted a review of the various modules and variables on the DHIMS2 platform. The purpose of the review was to identify the various features available and also to understand how information on the DHIMS2 platform could support a variety of decisions at the district, sub-district, and community level of the health system.

Data collection tools and interview guides were developed based on the 2011 version of Measure Evaluate Performance of Routine Information Systems Management (PRISM) tools [14]. The resulting tools were the district assessment form (see Additional file 1), the facility assessment form (see Additional file 2), the behavioral assessment form (see Additional file 3), the desk review guide (see Additional file 4), and the in-depth interview guide (see Additional file 5). During the data collection process, 10 indicators, along with an assessment scale, were used to assess the perceived capacity of participants to execute certain tasks on the DHIMS2 platform. The scale was 0 to 100. The higher the percentage, the higher the capacity of the respondent to perform the task, and vice versa. The 10 indicators were as follows;

- It is easy to manage Information Technology (IT) (EMIT)
- DHIMS2 is user-friendly software (DUFS)
- Ability to generate and follow the monthly reports in DHIMS2 (AGMR)
- Ability to make decisions using DHIMS2 data (ADMD)
- Ability to identify annual targets using DHIM2 data (AIAT)
- Ability to explain finding from DHIMS2 data (AEFD)
- Ability to compute trends from the DHIMS2 platform (ACTD)
- Ability to plot data on charts on the DHIMS2 platform (APDC)
- Ability to calculate rates from DHIMS2 data (ACRD)
- Ability to enter data accurately unto DHIMS2 (AEDA)

Four research officers administered the tools. The research officers were trained with the skills needed for data collection. A pilot study was conducted outside of the study area to pre-test the data collection tools.

Data were collected electronically using the REDCap platform (REDCap 8.3.2 -© 2018 Vanderbilt University) deployed by the Kintampo Health Research Centre (KHRC). The mobile version of REDCap was installed on four tablets used for data collection.

### Data processing and analysis

Working with the Data Collection Team, the Data Manager resolved the queries as and when the data was transferred to the KHRC REDCap database. Due to the near real-time transmission of data from the field, the query resolution and data collection were carried out simultaneously. STATA 14 was used to analyze the data. A combination of statistical tables, charts, and percentages was used to interpret the results.

### Ethical consideration

Ethical clearance was obtained from the institutional ethics review board of the Kintampo Health Research Centre. Written informed consent was obtained from each participant and a copy of their consent forms given to them. During the consenting process, the aims, objectives, and study procedures were thoroughly explained to all study participants. Participants were assured of the anonymity and confidentiality of the information they provide. They were also informed that participation in the study was voluntary. During the consenting process, three participants declined to be part of the study.

## Results

As earlier mentioned, a total of 107 health facilities in the Brong Ahafo region was selected for the study. Of 195 participants sampled, one of the selected DGHs declined to participate in the study. Also, one person from another DGH could not be contacted upon several visits. Also, data from 18 participants at the community level were excluded from analysis. These participants were in CHPS zones where there was no physical infrastructure to allow the required observations to be made during the assessment and desk reviews.

### Socio-demographic characteristics of respondents

Data were analyzed for 93 (53.7%) males and 78 (45.1%) females. Sixty-five (38.0%) of the respondents had at least undergraduate education, while 108 (62.0%) had post-secondary / high school credentials as their highest qualification (e.g. Diploma or Certificate in Nursing, Midwifery, Medical Assistant, Computing, etc.). Responses came from a wide variety of health professionals. The top three among were nurses and midwives (28.3%), community health officers (20.2%), and health information officers/biostatisticians (18.4%). A total of 89 (51.4%) respondents had 1 to 5 years of work experience in their current positions. Although 3 respondents at the district level had more than 15 years of experience, none of the respondents at the sub-district and community level had served in their current roles for more than 10 years at the time of this report.

### Modules and variables in the DHIMS2 platform

The DHIMS2 platform included variables for the collection of individual care delivery data on out-patient, admission, maternal and child health services, among others. The DHIMS2 platform also included variables for collecting data on public health events such as immunization campaigns, distribution of bed-nets, and outbreaks of disease, among others. Population estimates of the catchment areas of health facilities were also recorded in the DHIMS2 database. From an administrative point of view, the DHIMS2 platform had modules for the collection of human, equipment, and other resource capacity data from health facilities. The platform provided options for viewing analyzes generated from different datasets for decision support. These options ranged from simple bar graphs to geo-location-based visualizations and could compare different datasets. Due to the DHIMS2 platform’s modular nature, data analysis may be done either at the individual facility level, or by integrating data from multiple health facilities. Aggregation of data may also be performed based on the health system’s hierarchical structure as built into the software. Constraints on data access have also been correlated with the platform’s modular architecture.

### Utilization of DHIMS2 information in decision-making

**Fig. 4:**
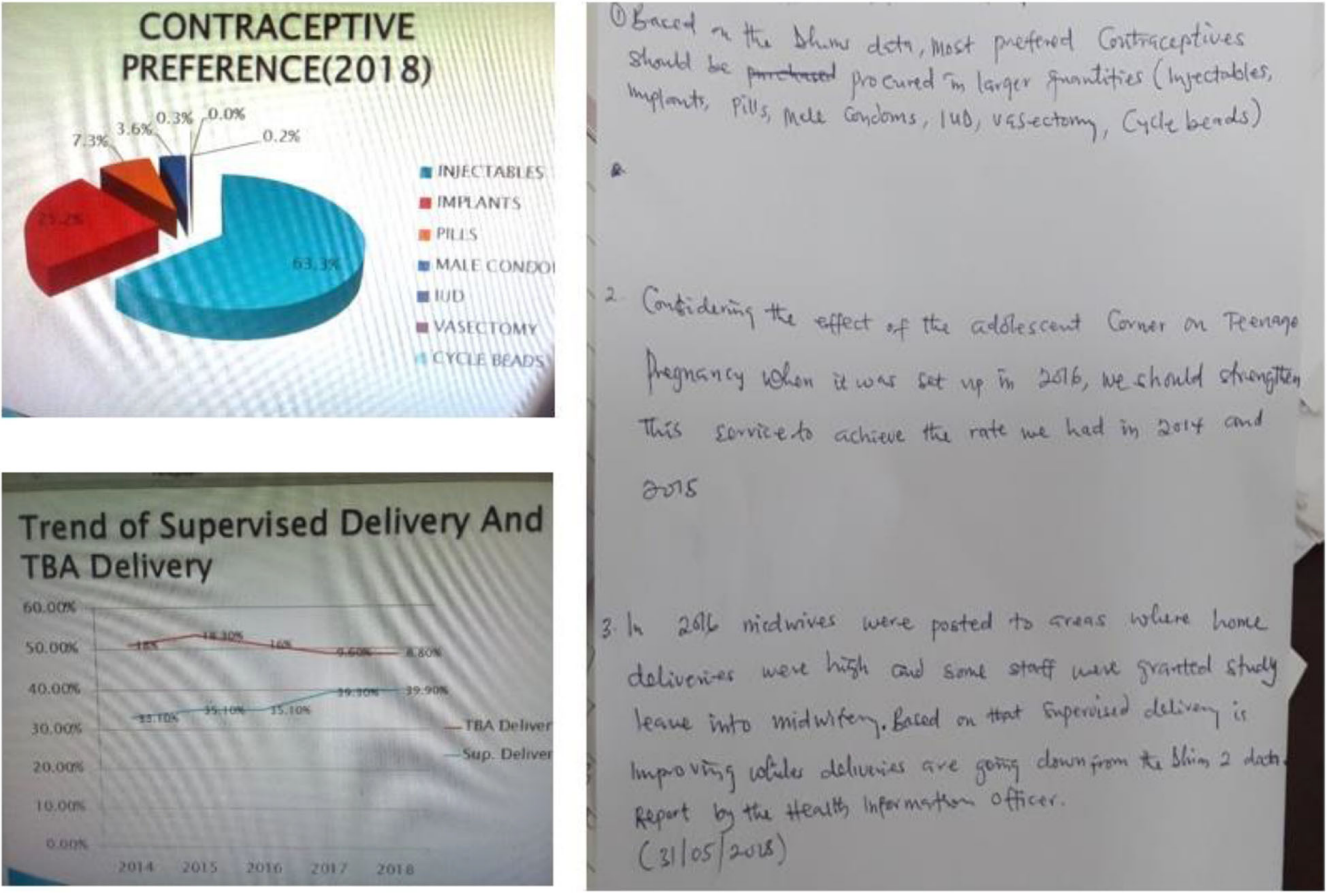
Example of how DHIMS2 data was used to support decision making at a DHMT. An excerpt from the desk review that shows an example of how a pie chart and a trend line chart from the DHIMS2 platform have been used to facilitate decision-making

From the desk review conducted, the utilization of DHIMS2 information in decision-making was examined. Specific areas of interest were whether the facilities organized routine meetings to take decisions and, if so, the forms of discussions about the DHIMS2 platform, action-oriented decisions taken based on findings from DHIMS2 data, and actions taken to promote the usage of the DHIMS2 platform. The detailed analysis of discussions, decision and actions were limited to documented proof from the last 3 months preceding the study. Further analysis was undertaken on the forms of action-oriented decisions informed by DHIMS2 platform data, and four groups emerged. Analysis of actions taken focused on directives and promotional activities that promoted the use of DHIMS2 data. To elaborate further on the assessment made from the desk review, an excerpt is presented in Fig. 4.

From the excerpt (Fig. 4), directives on the types of contraceptives to procure were based on a pie chart sourced from DHIMS2 data. Also, a trend line graph was used to compare institutionally supervised deliveries with deliveries by traditional birth attendants (TBA) over 5 years. A decision was made to strengthen some of the actions taken in 2016 that were believed to have contributed to the reversed trends of the delivery patterns. The last paragraph of the report excerpt from Fig. 3 describes how a decision to train and transfer midwives to places where deliveries by TBAs were high may have led to the changes in institutionally supervised delivery. Given similar scenario examples presented in other facilities, the results of the desk review of the use of DHIMS2 data for decision making are presented in Table 2.

**Table 2:** Utilization of DHIMS2 Data in Decision-Making

**Organizing decision meetings and having an action plan**										
	**DHMT**		**DGH**		**Sub-District**		**Community**		**Total**	
	**(*N*** **= 12)**		**(*N*** **= 10)**		**(*N*** **= 29)**		**(*N*** **= 38)**		**(*N*** **= 89)**	
	**n**	**%**	**n**	**%**	**n**	**%**	**n**	**%**	**n**	**%**
Managerial meetings	12	100.0	10	100.0	24	82.8	21	55.3	67	75.3
Meetings are routine	12	100.0	10	100.0	23	79.3	20	52.6	65	73.0
Meeting held in last 3 months	12	100.0	9	90.0	23	79.3	21	55.3	65	73.0
**Discussions on DHIMS2 information in meetings held within the last three months**										
	**DHMT**		**DGH**		**Sub-District**		**Community**		**Total**	
	**(N = 12)**		**(*N*** **= 9)**		**(*N*** **= 23)**		**(*N*** **= 21)**		**(*N*** **= 65)**	
	**n**	**%**	**n**	**%**	**n**	**%**	**n**	**%**	**n**	**%**
Data management issues	10	83.3	9	100.0	16	69.6	21	100.0	56	86.2
Findings from DHIMS2 data	10	83.3	5	55.6	14	60.9	4	19.0	33	50.8
Action-oriented decisions from DHIMS2 data findings	11	91.6	9	100.0	14	60.9	5	23.8	39	60.0
Follow-ups from previous decisions	11	91.6	7	77.8	14	60.9	5	23.8	37	56.9
Referrals of issues for action	7	58.3	6	66.7	7	30.4	5	23.8	25	38.5
**Decisions for actions based on DHIMS2 information within the last three months**										
	**DHMT**		**DGH**		**Sub-District**		**Community**		**Total**	
	**(N = 12)**		**(N = 9)**		**(N = 23)**		**(N = 21)**		**(N = 65)**	
	**n**	**%**	**n**	**%**	**n**	**%**	**n**	**%**	**n**	**%**
Performance recognition and role/responsibility revision	6	50.0	7	77.8	13	56.5	5	23.8	31	47.7
Shifting/mobilization of resources	5	41.7	5	55.6	10	43.5	4	19.0	24	36.9
Advocacy for more resources	5	41.7	7	77.8	12	52.2	5	23.8	29	44.6
Formation/revision of policies/strategies	4	33.3	8	88.9	10	43.5	5	23.8	27	39.1
**DHIMS2 usage & promotion activities within the last three months**										
	**District directorate**		**District Hospital**		**Sub-District**		**Community Facilities**		**AllFacilities**	
	**(N = 12)**		**(N = 9)**		**(N = 23)**		**(N = 21)**		**(N = 65)**	
	**n**	**%**	**n**	**%**	**n**	**%**	**n**	**%**	**n**	**%**
Issued directives to encourage usage of DHIMS2 data	7	58.3	7	77.8	8	34.8	8	38.1	30	46.2
Publishing example of DHIMS2 data usage	4	33.3	4	44.4	3	13.0	3	14.3	14	21.5
Feedback on actions based on DHIMS2	6	50.0	6	66.7	10	43.5	10	47.6	32	49.2

From the results in Table 2, all 22 facilities at the district level had routine management meetings for decision-making. At the sub-district level 82.8% of health centers conducted routine decision-making meetings, while at community level 55.3% of facilities had routine meetings. In the last 3 months, all 12 DHMTs had held a management meeting, while 9 out of 10 DGHs had held the same meetings. At sub-district level 23 (79.3) health centers also held a management meeting within the last 3 months, while 21 (55.3%) community-level facilities had also held the same meetings. At district, sub-district and community levels, all facilities that had held a management meeting during the last 3 months had proof of a discussion, decision and/or action taken based on the DHIMS2 platform.

During the desk review, it emerged that 11 (91.6%) of the 12 district-level DHMTs had reported decisions in their minutes aimed at taking actions based on DHIMS2 data findings, which closely correlated with DGH results where all 9 had proof of action-oriented decisions informed by DHIMS2 data. Discussions at the sub-district level on DHIMS2 data management issues were observed in 16 out of 23 health centers. The records of 14 of the sub-district facilities had evidence of decisions aimed at taking actions based on finding from DHIMS2. Results from the desk review showed that all community-level facilities had records of discussions related to data management issues in DHIMS2 data, it was interesting to realize that the remaining discussions on findings from the DHIMS2 data, action-oriented decisions, problems that should be assigned to supervisors and follow-ups from previous meeting discussions were observed in less than 25% of the community level facilities. The referral and follow-up discussions had mainly revolved around the technical and logistic challenges of data entry on the DHIMS2 platform.

As for the types of action-oriented decisions, a total of 31 (47.7%) of the 65 facilities that held meetings during the last 3 months had records of the decision to recognize performance and/or review staff responsibilities. A proportion of 5 (41.7%) out of 12 DHMTs had addressed shifting and/or mobilizing resources based on comparisons made with DHIMS2 knowledge during the last 3 months. At the community level this was much lower; 4 (19.0%) of the 21 facilities had these discussions. Interestingly, in the last 3 months, 8 (88.9%) of the 9 DGHs had evidence of decisions to review/update policies and/or strategies based on DHIMS2 data findings. Across all 65 facilities, 27 (39.1%) had proof of discussions to review policies/strategies based on DHIMS2 information. From a general point of view, DGHs have shown a leading role in the discussion of decisions to act based on DHIMS2 information while the community level had the lowest proportion of 5 (23.8%) for the 21 facilities that held routine meetings.

With regards to actions aimed at promoting the use of the DHIMS2 platform, results from Table 2 indicate that among facilities that had held meetings in the last 3 months, 7(58.3%) of 12 DHMTs, 7 (77.8%) of 9 DGHs, 8 (34.8%) of 23 health centers and 8(38.1%) of 21 community-level facilities had issued directives on the usage of DHIMS2 data. Concerning the publishing of examples of using DHIMS2 information, evidence was available for 4 (33.3%) of 12 DHMTs, 4 (44.4%) of 9 DGHs, 3 (13.0%) of 23 sub-district facilities and 3(14.3%) of 21 community-level facilities.

### The perceived capacity to perform certain tasks using the DHIMS2 platform

This section presents results from the 10 selected indicators used to evaluate the perceived capacity of decision-makers in performing tasks on the DHIMS2 Platform. The results were presented by the level of the health system being studied. Results of the DHMTs for the district level were segregated from those of the DGHs.

**Fig. 5:**
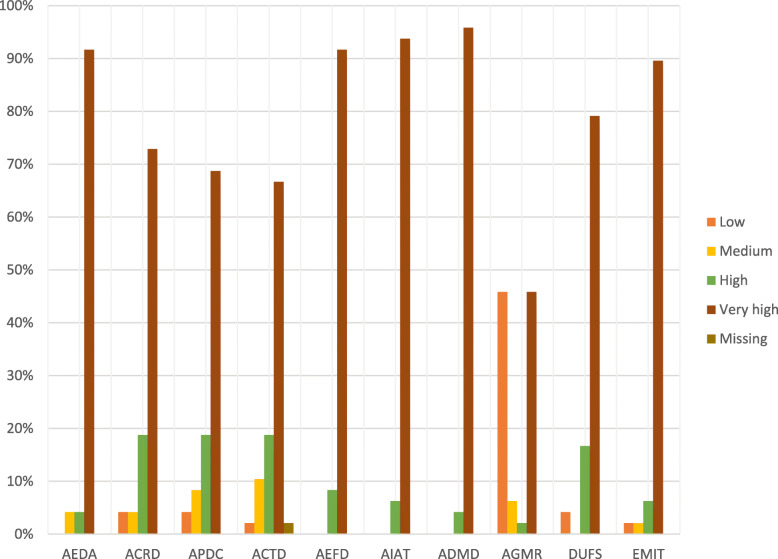
Perceived Capacities of DHMT Participants at District Level to use the DHIMS2 Platform. For simplicity, the abbreviations in the data collection section under Methods were used in place of the indicators to label the charts. The assessment scale was organized into four categories. Ranges from 0 to 30 were classified as low capacity, ranges from 40 to 50 were classified as medium capacity, range 60–70 was classified high capacity and range 80–100 as very high capacity

Figure 5 indicates the perceived capacity of DHMT participants to use DHIMS2. For almost all of the indicators, over 70% of the 48 respondents ranked themselves at very high capacity. In terms of ease of generation and following the monthly reports in DHIMS2, the very low and very high capacity groups each had a 45.83% share of participants. Concerning the ability to make decisions using DHIMS2 data, more than 95% of the participants rated their capacity within the very high group.

**Fig. 6:**
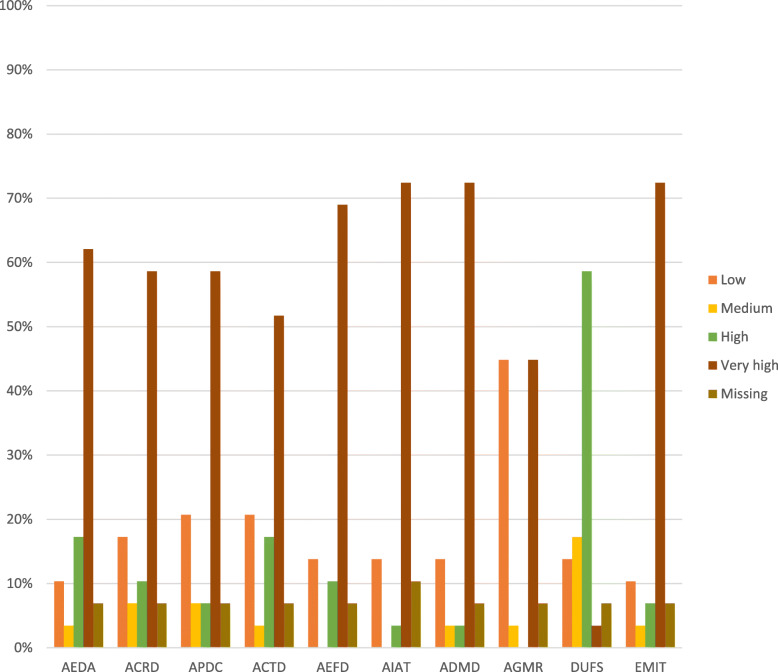
Perceived Capacities of DGH Participants at District Level to use the DHIMS2 Platform. For simplicity, the abbreviations in the data collection section under Methods were used in place of the indicators to label the charts. The assessment scale was organized into four categories. Ranges from 0 to 30 were classified as low capacity, ranges from 40 to 50 were classified as medium capacity, range 60–70 was classified high capacity and range 80–100 as very high capacity

Of the 29 participants interviewed as presented in Fig. 6, a proportion of 72.41% rated their ability to identify annual targets using DHIM2 data, explain DHIMS2 data findings and handle IT easily within the very high capacity group. More than 58% of DGH participants also rated their skill in the very high capacity category to calculate rates and plot data. Similar to the patterns among DHMT participants, each of the very low and very high capacity groups had a 44.83% share of DGH participants in terms of ease of generation and following the monthly DHIMS2 data.

**Fig. 7:**
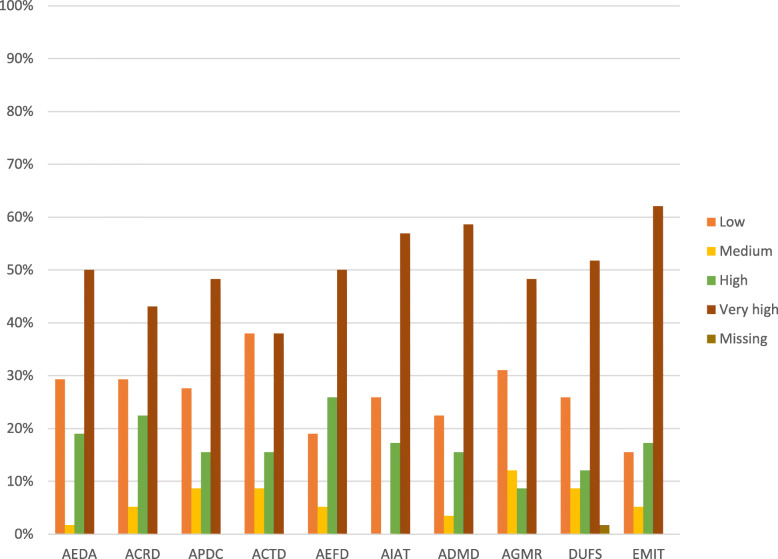
Perceived Capacities of Sub-District Level Participants to use the DHIMS2 Platform. For simplicity, the abbreviations in the data collection section under Methods were used in place of the indicators to label the charts. The assessment scale was organized into four categories. Ranges from 0 to 30 were classified as low capacity, ranges from 40 to 50 were classified as medium capacity, range 60–70 was classified high capacity and range 80–100 as very high capacity

Out of the responses received from 58 sub-district participants, as shown in Fig. 7, a proportion of 56.90% rated their ability to identify annual targets using DHIM2 data, explain findings from DHIMS2 data and manage IT easily at very high capacity. The ability to calculate rates (43.10%) and the ability to plot data (48.28%) were two other indicators that had over 40% of sub-district level participants ranking at very high capacity. With regards to the ease of generating and following the monthly reports in DHIMS2, 31.03% sub-district level participants ranked their perceived capacities at very low. Also, 29.31% of the respondents ranked their ability to enter data accurately unto DHIMS2 at very low capacity.

**Fig. 8:**
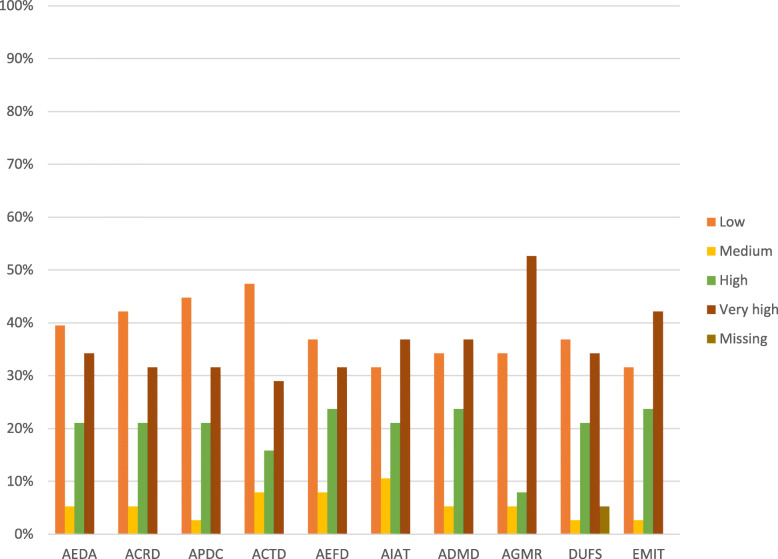
Perceived Capacities of Community-Level Participants to use the DHIMS2 Platform. For simplicity, the abbreviations in the data collection section under Methods were used in place of the indicators to label the charts. The assessment scale was organized into four categories. Ranges from 0 to 30 were classified as low capacity, ranges from 40 to 50 were classified as medium capacity, range 60–70 was classified high capacity and range 80–100 as very high capacity

As shown in Fig. 8, more than 30% of participants at the community level considered themselves to have very high capacity in their ability to define annual targets using DHIM2 data, explain findings from DHIMS2 data and easily manage IT. Also, 31.6% ranked their perceived ability to calculate rates and plot data within the very high category. It was also interesting to note that 34.2% of participants at the community level indicated very low capacity in generating and following the monthly reports in DHIMS2. Also, 39.5% of the respondents ranked their ability to enter data accurately unto DHIMS2 in the very low capacity category.

## Discussion

We precede the discussions with a summary of some evaluation studies that have been performed on how the use of HIS data in certain African countries is feeding into decision making. At the beginning of 2009, a cross-sectional study was conducted among populations selected from district health offices, health centers and health posts in Ethiopia to evaluate the utilization of a health management information system (HMIS) [15]. In that study, particular attention was paid to factors associated with the utilization of HMIS. From that study, the rate of utilization of information from the HMIS was far below national expectations. That study also reported that although an average of 57% of participants tried to transform data into information, 32.1% used the information for decision making. Other factors identified include poorly coordinated processes and multiple information formats that were not fully automated. That study also discovered a perception among participants that utilization of information was for the higher levels, limiting their responsibility to pushing data. Reasons ascribed to this were primarily due to heavy emphasis on data submission deadlines and weak feedback mechanisms from supervisory control activities. Another cross-sectional study in Ethiopia assessed the utilization of health information systems which special emphasis on HIV/AIDS data. In this study, the general utilization of information from the health information system was 22.5%. The study also identified influencing factors similar to those previously identified the forgoing literature reviewed.

Findings from the review of the DHIMS2 platform suggested that the platform provided support for level to level decision-making in the health system. Health facilities at the community levels could, for instance, generate seasonal trend analyses of patient attendance, total admission and morbidity from case-based individual care delivery service data to facilitate decision on which diagnostic kits and/or resources to strongly advocate for and the timing for advocating for the resources. Community-level public health outreach programme organized by the community health facilities could also use the catchment population estimates as denominators for estimating the coverage of the public health events and deciding how personnel responsibilities could be efficiently mobilized and/or shifted to achieve expected targets.

The district-level could generate aggregated views of data across health facilities within their administrative boundaries and make comparisons to achieve more targeted interventions, focused training, and efficient supervision. Administrators at the district level could also use information from the DHIMS2 platform to effectively monitor resource allocation efforts, identify gaps, and guide how subsequent limited resources would be equitably distributed.

In the study area, the level of utilization of information in decision-making among the studied facilities at all three levels ranged from 96% at the district levels to 14% at the community-level health facilities. Comparing this analysis with the aforementioned research studies conducted in Ethiopia, data utilization was higher in DHIMS2. Also in the Ethiopian study, poorly coordinated processes and data multiple information formats in the HIMS were mentioned as factors that had negative impacts on utilization. Given these influencing factors identified by the studies conducted in Ethiopia would suggest that the nature of the DHIMS2 platform that allows data entry, processing, and harmonization in a unified manner across all health facilities has significantly improved data presentation in a way that has encouraged usage over the years.

From a desk review of content relating to discussions on data quality and data management. There was evidence of elaborate deliberations that were sufficient to suggest a commitment to improving the quality of DHIMS2 data across all 12 districts. However, overall discussions were skewed towards data quality and data management issues and lesser to gaining insights from data for decision-making. This was predominantly at the sub-district and community level facilities where less evidence was observed on discussions on findings and making comparisons from the DHIMS2 data, decisions for actions based on comparisons made, and definite actions (advocacy, promotional, directives, etc.) resulting from discussions. Studies from Ethiopia also show similar trends and realized that lower-level facilities were primarily engaged in pushing data to higher-level facilities to analyze. Notwithstanding this, the proportions of lower-level facilities utilizing DHIMS2 data in the implementation of activities to promote the usage of DHIMS2 were relatively substantial.

The results also presented a typical scenario on how some facilities developed their action-oriented decisions from findings they observed in DHIMS2. Undeniably, the argument that decisions from previous years made to encourage subsequent actions taken appeared to yield results in reversing the trends of home deliveries may be weak based on the evidence presented. However, this evidence of clear linkages drawn between discussion of findings and decisions for actions are encouraging.

At the DHMTs, results from the perceived ability of participants in performing tasks on the DHIMS2 platform suggested that significant proportions of respondents had high capacity in understanding the data from the platform to inform decision making. However, a significant proportion found the generation and following of monthly reports relatively complex. At the district/referral hospitals, results suggested that perceived capacity amongst respondents in performing tasks on the DHIMS2 generally did not rank as high as what was observed amongst respondents at the DHMT. Over 70 % of respondents ranked highly their capacity in utilizing DHIMS2 data to identify annual targets and make decisions. Again, a significant proportion found the generation and following of monthly reports relatively complex.

The DHIMS2 platform effectively integrates data from multiple health facilities at a national scale to support decision making. Reliance on only facility-based data for health systems decision making is not adequate due to the variations in health-seeking practices and lack of data representativeness of facility-based data especially in LMICs [16]. The platform will benefit from data from population-based surveys at the household level especially among those who do not seek care in health facilities [17].

## Recommendations

The DHIMS2 platform was implemented to ensure that health policy formulation in Ghana is guided by reliable and timely evidence. Non-use of DHIMS2 data may undermine this purpose, which could perpetuate the inefficiencies in the allocation of resources and delay progress towards achieving health sector objectives and performance targets. Moreover, in implementing ICT related policies and programs substantial resources are sunk into the design and implementation as well as infrastructure for operational purposes. To justify such investments, it is essential to take steps in ensuring efficient utilization to realize the full benefits of such expensive investments. This study raised key issues that could feed into policy suggestions and options for enhancing the effective utilization of the DHIMS2 health information system for decision.

The supervisory role of the district and sub-district leadership must increase in terms of frequency of visits and assisting facilities on how to derive insights from DHIMS2 data and form actions to improve the lives of their catchment populations. This is especially concerning supervision at the community level facilities.

District and sub-district leaderships should initiate periodic newsletters and publications that show examples of how findings from DHIMS2 have been translated into decisions for actions. Facilities at the lower levels should be pushed to publish similar examples in the newsletters. Such an initiative could build the abilities and confidence of staff in explaining and applying findings.

Considering issues of staff shifting and transfers, skills development and training on the utilization of DHIMS2 could be considered an option in health training institutions or during the national service period of emerging staff. This could ensure that newly recruited staff have already developed some skill in the use of DHIMS2. This however, should not preclude periodic in-service training. An abridged version of the training manual should by policy be displayed on a notice board that is easily accessible to all health staff in each health facility.

Concerning the sporadic introduction of new form formats, the technical team responsible for introducing new forms should prepare and attach a reference manual (possibly electronic) as a user guide to the DHIMS2 platform.

## Conclusion

This study examined whether DHIMS2 information was being used in decision making, explored organizational, technical and behavioral factors that affected unit heads in using DHIMS2 data to inform a decision, identified barriers and enablers to the use of DHIMS2 in decision making and solicited recommendations from healthcare managers for enhancing their utilization of the DHIMS2 information in health decision making. Drawing from results, full integrations of DHIMS2 information in their decision-making processes is yet to be achieved. The absence of a manual for guiding the use of DHIMS2, the lack of frequent training at the sub-district and community levels, and internet access were challenges for both the district and sub-district levels.

Information from this research would be useful in improving how unit heads at the district, sub-district, and community health units (health centers and CHPS) use DHIMS 2 data to inform decisions to improve health service delivery in their areas. Information from this research would also be useful to regional and national heads, the DHIMS 2 administrators, and Ghana health service. These institutions could use this information for further training and intensify supervision. The information would also be relevant to the government of Ghana and donors and international bodies who are investing in the system and are interested in evidence-based decision making in the public health sector. The outcome of this study could also serve as a guide to other countries that are interested in promoting the use of routine health information systems to inform decision making.

## Supplementary information

**Additional file 1.** District Assessment Form. The district assessment form is quantitative interview guide used to collect data from participants who were selected from the district health management team.

**Additional file 2.** Facility Assessment Form. The facility assessment form is a quantitative interview guide used to collect data from participants who were selected from health facilities at the district, sub-district and community levels.

**Additional file 3.** Behavioral Assessment Form. The behavioral assessment form is a quantitative interview guide used to collect data from all selected participants during the study.

**Additional file 4.** Desk Review Guide. The desk review guide is an observational interview guide that was used to guide the review of documented evidence all three levels of the district health system during the study.

**Additional file 5.** In-depth Interview Guide. The in-depth interview guide is a qualitative interview guide that was used to collect qualitative data for the purpose of the study. This file is in PDF format

## Data Availability

The datasets generated and/or analyzed during the current study are not publicly available due to institutional policies but are available from the corresponding author on reasonable request.

## Abbreviations

ACRD: Ability to calculate rates from DHIMS2 data
ACTD: Ability to compute trends from the DHIMS2 platform
ADMD: Ability to make decisions using DHIMS2 data
AEDA: Ability to enter data accurately unto DHIMS2
AEFD: Ability to explain finding from DHIMS2 data
AGMR: Ability to generate and follow the monthly reports in DHIMS2
AIAT: Ability to identify annual targets using DHIM2 data
APDC: Ability to plot data on charts on the DHIMS2 platform
CHPS: Community-based health planning and services
DGH: District Government Hospital
DHIMS2: District health information management system (version 2)
DHIS2: District health information system (version 2)
DHMT: District health management team
DUFS: DHIMS2 is user-friendly software
EMIT: It is easy to manage Information Technology
GHS: Ghana Health Service
HIS: Health information system
HMIS: Health information management system
IT: Information Technology
KHRC: Kintampo Health Research Centre
LIMC: Low and middle-income country
PRISM: Performance of Routine Information System Management
TBA: Traditional birth attendant
