# Supplementary material for: Utilization of the national cluster of district health information system for health service decision-making at the district, sub-district and community levels in selected districts of the Brong Ahafo region in Ghana
Source: BMC Health Serv Res. 2020 Jun 6;20:514. doi: 10.1186/s12913-020-05349-5 (PMC7275484; doi:10.1186/s12913-020-05349-5)
Supplement: Supplementary file 1 — Additional file 1. District Assessment Form. The district assessment form is quantitative interview guide used to collect data from participants who were selected from the district health management team. [file 12913_2020_5349_MOESM1_ESM.pdf]

KINTAMPO HEALTH RESEARCH CENTRE<br>DISTRICT ASSESSMENT FORM<br>DHIMS 2 EVALUATION STUDY 06/07/2017	FORM NO	FORMNO

1. BASIC INFORMATION:

1.1. Village code and name? ………………		V_code
1.2. Name of facility?		Fac_name
1.3. Date of visit……………………		Day_visit

1.4. Type of facility?

1. Hospital	2.Health Centre	3. Private Clinic	4. CHPS	Type_hf
5. DHMT facility (eg Kintampo RCH)	6. Other, specify….			

1.5. ENTER FACILITY CODE: ………………………………………………… Hf_code

1.6 Respondent's name ……… r_name

1.7. Sex of respondent ………

1. Male	2.Female	R_sex

1.8. Religion? .....

1. Christian	2.Muslim	3. Traditional	4. Other, specify	R_religion

1.9. Marital status? .....

1.Married	2. Never married/single	3. Divorced	4. Co-habiting	R_maristatus

1.10. Highest educational level? ....

1. Certificate	2. Diploma	3. Gradu at e d egree/above	R_edulevel

1.11. What is your profession?

1. Med. doctor	2.Med. assistant	3. RN/PHN	4. Lab tech	5. Pharmacist	R_profession
6. Biostatistician	6. Nutritionist	7. CHO	8. IT personnel	9. Other specify………	

1.12. What is your job title?

1. Director	2.Administrator	3. Matron	4. In-charge	R_jobtitle
5. Head of department	6. Program/Project Coordinator	7. Other, specify		

1.13. How long have you been working in this facility? ....

Year			Years_work
Month			Months_work
Weeks			Weeks_work
Days			Days_work

1.14. How long have you been working in your current capacity? ....

Year			Cap_years
Month			Cap_months
Weeks			Cap_weeks
Days			Cap_days

SECTION 2

2.1. Where/whom do you report to?...

1.Region/ National	2.Direct DHMT	2. District	3. Sub-district/Health centre	Report_ends

2.2. What is the number of facilities that are supposed to be reporting to (enrolled in) DHIMS 2? ………..			Fac_number
2.3.What is the number of facilities in the district that are actually reporting to (enrolled in) DHIMS 2? ………			Actual_report
2.4. Does the district have a record of people who receive monthly report data by a certain deadline after receiving monthly reports from the facilities? ………………………..	1. Yes	2. No	Month_report
2.5. Does the district have a record of submitting data on time to regional and/or national levels?	1. Yes	2. No	sub_records

2.6. Does DHIMS2 produce the following?

2.6a.Calculate indicators for each facility catchment area?	1. Yes	2. No	Cal_indicators
2.6b.Data summary report for the district?	1. Yes	2. No	Data_summary
2.6c.Comparisons among facilities? ……………………………………………………………….	1. Yes	2. No	Comp_fscilities
2.6d.1.Comparisons with district/national target?	1. Yes	2. No	Comp_dist/nat
2.6e. Comparisons among types of services coverage?	1. Yes	2. No	Comp_services
2.6f.Comparisons of data over time (monitoring over time)?	1. Yes	2. No	Comp_dataovert

2.7. Do you have a procedure manual? ………………………………………………..................	1. Yes	2. No	Proce_manual
2.8. Do you think the monthly report form is complex and difficult to follow?	1. Yes	2. No	Form_complex
2.9. Do you find the data software to be user-friendly?	1. Yes	2. No	User_friendly
2.10. Do you find that information technology is easy to manage?	1. Yes	2. No	Tech_easyuse
2.11. Do you think that information system design provides a comprehensive picture of health system performance?	1. Yes	2. No	Syt_perform

2.12. Does the information technology (Land Area Network-LAN or wireless network) exist to provide access to information to all district managers and senior management?

1. Yes partially	2. Yes completely	3. No	Netwk_access

3. USE OF INFORMATION DISTRICT ASSESSMENT FORM

3.1. Does this district office compile DHIMS 2 Data submitted by facilities? …………	1. Yes	2. No	Distoff_compile
3.2. Does the district issue any report containing DHIMS 2 information? …………….	1. Yes	2. No	Dist_issueinfo

If Q3.2 is yes, please list reports that contain data/information generated through the DHIMS 2. Please indicate the frequency of these reports and the number of times the reports actually were issued during the last 12 months. Please confirm the issuance of the report by counting them and putting the number in column 3.

3.3	1. Title of report	2.No. of times this report is supposed to be issued per year	3. No. of times that report are actually issued for the last 12 months	
3.3a.				Report_one
3.3b.				Report_two
3.3c.				Report_three
3.3d.				Report_four
3.3e.				Report_five
3.3f				Report_six
3.3g.				Report_seven

3.4. Did the district office send a feedback report using DHIMS 2 information to facilities during the last three months? ……………………………………………………			Feedback_fac

4. DISPLAY OF INFORMATION

4.1	Does the district office display the following data? Please indicate the types of data displayed and whether the data are updated for the last reporting period.<br><br>If no go to Q4.2					
	1.Indicator	2.Type of display (Please tick)		3. Updated		
4.1a.	Related to mother health	Table		1.Yes	2. No	Rel_motherH
		Graph/Chart				
		Map				
4.1b.	Related to child health	Table		1.Yes	2. No	Rel_childH
		Graph/Chart				
		Map				
4.1c.	Facility Utilization	Table		1.Yes	2. No	Rel_facutiliza
		Graph/Chart				
		Map				
4.1d.	Disease surveillance	Table		1.Yes	2. No	Rel_diseaseS
		Graph/Chart				
		Map				

4.2. Does the office have a map of the catchment area?..................	1. Yes	2. No	Area_map
4.3. Does the office display a summary of demographic information such as population by target group(s)?	1. Yes	2. No	Demo_infor
4.4. Is feedback quarterly, yearly or any other report on DHIMS 2 data available, which provides guidelines/recommendations for actions?	1. Yes	2. No	Feedback_time

If Q4.4 is 2 No, go to Q5.1

4.5.	If yes to 4.4, what kinds of decisions are made in reports of DHIMS 2 data/information for actions? Please check types of decision based on types of analysis present in reports.			
	Types of decisions based on types of analysis			
4.5a.	Appreciation and acknowledgement based on number/percentage of facilities showing performance within control limits over time (month to month comparisons)	1. Yes	2. No	Appre_ackno
4.5b	Mobilization/shifting of resources based on comparison by facilities	1. Yes	2. No	Shift_resource
4.5c.	Advocacy for more resources by comparing performance by areas (sub-districts, cities, villages), human resources and logistics	1. Yes	2. No	Advoc_resource
4.5d.	Development and revision of policies by comparing types of services	1. Yes	2. No	Devel_policies

5. DISCUSSION AND DECISIONS ABOUT USE OF DHIMS2 INFORMATION

5.1. Does the district office have routine meetings for reviewing managerial or administrative matters?........	1. Yes	2. No	Routine_meet

5.2. How many times did the meeting take place during the last three months?

1. Weekly	2. After every two weeks	3. Monthly	4. Quarterly	5. No schedule	Sche_meeting

5.3. How many times did the meeting take place during the last three months?

1. 12 times	2. Between 7 and 11	3. 6 times	4. Either 4 or 5	5. 3 times	Meeting_times
6. 2 times	7. 1 time	8. None			

5.4	Is an official record of management meetings maintained?	1. Yes	2. No	Mgt_mrecord
5.5	If yes, please check the meeting records for the last three months to see if the following topics were discussed:<br><br>If no, go to Q6.1			

5.5a.	Management of DHIMS 2, such as data quality, reporting, or timeliness of reporting?..	1. Yes, observed	2. No	Mgt_timelines
5.5b	Discussion about DHIMS 2 findings such as patient utilization, disease data, or service coverage, or medicine stock out?.......	1. Yes, observed	2. No	Discuss_dhims
5.5c.	Have they made any decisions based on the above discussions?.....	1. Yes, observed	2. No	Any_decision
5.5d.	Has any follow-up action taken place on the decisions made during the previous meetings?...............................	1. Yes, observed	2. No	Follow-up
5.5e.	Are there any DHIMS 2 related issues/problems referred to regional/national level for actions?	1. Yes, observed	2. No	Prob_referred

6. PROMOTION AND USE OF DHIMS 2 INFORMATION AT DISTRICT/HIGHER LEVEL

6.1. Did district annual action plan showed decisions based on DHIMS 2 information?..	1. Yes	2. No	Action_plan
6.2. Did records of district office of last three months show that district/senior management issued directives on use of information……	1. Yes	2. No	Mgt_directives
6.3. Did district/national DHIMS 2 office publish newsletter/report in last three months showing examples of use of information………	1. Yes	2. No	Pub_reports
6.4. Does documentation exist showing the use of information for various types of advocacy?....................................	1. Yes	2. No	Doc_infoexist
6.5. Does the district staff meeting records show attendance of persons in charge of the facilities for discussion on DHIMS 2 performance?........	1. Yes	2. No	Incharge_meet
6.6. Does the district have annual action plan?.................	1. Yes	2. No	Annual_rept

**7.** Form checked and certified by?...	1. Yes	2. No	FW_FS

END OF FORM. CHECK YOUR FORM AND THANK THE RESPONDENT
