# Supplementary material for: Utilization of the national cluster of district health information system for health service decision-making at the district, sub-district and community levels in selected districts of the Brong Ahafo region in Ghana
Source: BMC Health Serv Res. 2020 Jun 6;20:514. doi: 10.1186/s12913-020-05349-5 (PMC7275484; doi:10.1186/s12913-020-05349-5)
Supplement: Supplementary file 2 — Additional file 2. Facility Assessment Form. The facility assessment form is a quantitative interview guide used to collect data from participants who were selected from health facilities at the district, sub-district and community levels. [file 12913_2020_5349_MOESM2_ESM.pdf]

KINTAMPO HEALTH RESEARCH CENTRE<br><br>FACILITY ASSESSMENT FORM<br><br>DHIMS 2 EVALUATION STUDY 06/07/2017	FORM NO	FORMNO

1. BASIC INFORMATION:

1.1. Village code and name? ………………			V_code
1.2. Name of facility? ………………………………			Fac_name
1.3. Date of visit……………………			Day_visit

1.4. Type of facility?

1. Hospital	2.Health Centre	3. Private Clinic	4. CHPS	Type_hf
5. DHMT facility (eg Kintampo RCH)		6. Other, specify….		

1.5. ENTER FACILITY CODE: ………………………………………………… Hf_code

1.6. Respondent Name R_name

1.7. Sex of respondent ………	1. Male	2.Female	R_sex

1.8. Religion? …..	1. Christian	2.Muslim	3. Traditional	4. Other, specify..	R_religion

1.9. Marital status?	1.Married	2. Never married/single	3. Divorced	4. Co-habiting	R_maristatus

1.10. Highest educational level? …	1. Certificate	2. Diploma	3. Graduate degree/above	R_edulevel

1.11. What is your profession?

1. Med. doctor	2.Med. assistant	3. RN/PHN		4. Lab tech	5. Pharmacist	R_profession
6. Biostatistician	6. Nutritionist	7. CHO	8. IT personnel	9. Other specify………		

1.12. What is your job title?

1. Director	2.Administrator		3. Matron	4. In-charge	R_jobtitle
5. Head of department		6. Program/Project Coordinator	7. Other, specify		

1.13. How long have you been working in this facility? ………..

Year			Years_work
Month			Months_work
Weeks			Weeks_work
Days			Days_work

1.14. How long have you been working in your current capacity? ………

Year			Cap_years
Month			Cap_months
Weeks			Cap_weeks
Days			Cap_days

SECTION 2. USE OF DHIMS2 INFORMATION

2.1. Does this facility compile DHIMS 2 Data? …………	1. Yes	2. No	Distoff_compile
2.2. Does the facility compile any report containing DHIMS 2 information? If **NO**, go to 2.3	1. Yes	2. No	Dist_issueinfo

2.2.1 If Q2.2 is **YES**, please list reports that contain data/information generated through the DHIMS 2.

Please indicate the frequency of these reports and the number of times the reports actually were issued during the last 12 months. Please confirm the issuance of the report by counting them and putting the number in column 3.

	1. Title of report	2.No. of times this report is supposed to be issued per year	3. No. of times that report are actually issued for the last 12 months
2.2.1 a.			
2.2.1 b.			
2.2.1 c.			
2.2.1 d.			
2.2.1 e.			
2.2.1 f			

2.3. Does the facility display the following data? Please indicate types of data displayed and whether the data have been updated for the last reporting period……	1. Yes	2. No	Fac_display

If NO, go to 2.4

1. Indicator	2. Type of display (Please tick)		3. Updated		
2.3.1a Related to maternal health	Table		1. Yes	2. No	Mat_Health
	Graph/Chart				
	Map/Other				

2.3.1b Related to child health	Table		1. Yes	2. No	
	Graph/Chart				
	Map/Other				Chi_Health
2.3.1c Facility utilization	Table		1. Yes	2. No	
	Graph/Chart				
	Map/Other				Fac_Util
2.3.1d Disease surveillance	Table		1. Yes	2. No	
	Graph/Chart				
	Map/Other				Dis_Surv

2.4. Does the facility have a map of the catchment area?	1. Yes	2. No	Map_Cat
2.5. Does the office display a summary of demographic information such as population by target group(s)?	1. Yes	2. No	Sum_Demo
2.6. During the last three months, did the facility receive any feedback report from district office on their performance?	1. Yes	2. No	Faci_Feedback
2.7. Is feedback, quarterly, yearly or any other report on DHIMS 2 data available, which provides guidelines/ recommendations for actions?...	1. Yes	2. No	Rep_data

If **No**, go to 2.8

2.7.1. If you answered **Yes** to question 2.8, what kinds of action-oriented decisions have been made in the reports (based on DHMIS 2 data)? Please check the boxes accordingly? ……………………………

2.7.1a Review strategy by examining service performance target and actual performance from month to month? …………………………………………………………………….	1. Yes	2. No	Rev_Perf
2.7.1b. Review facility personnel responsibilities by comparing service targets and actual performance from month to month? ……………………………………………………….	1. Yes	2. No	Rev_Resp
2.7.1c. Mobilization/shifting of resources based on comparison by services………	1. Yes	2. No	Mob_Serv
2.7.1d. Advocacy for more resources by showing gaps in ability to meet targets……	1. Yes	2. No	Advo_Gaps
2.8. Does the facility have routine meetings for reviewing managerial or administrative matters?	1. Yes	2. No	

If NO, go to 2.9……………….

2.8.1 If **YES**, how frequently is the meeting supposed to take place?

a. Weekly	b. After every two weeks	c. Monthly	d. Quarterly	e. No schedule	Rou_Freq

2.9. How many times did the meeting actually take place during the last three months? ....

a.12	b.7-11	c.6	d. 4 or 5	e. 3	d.2	e.1	f. none	Rou_months

2.10. Is an official record of management meetings maintained?	1. Yes	2. No	Rec_Manag

If no, go to 2.11……………………..

2.10.1 If yes, please check the meeting records for the **last three months** to see if the following topics were discussed:…………………..

2.10.1 a. Management of DHIMS 2, such as data quality, reporting, or timeliness of reporting……………………………………………….	1.Yes, observed	2. No	Mana_DHIMS 2
2.10.1 b. Discussion on DHIMS2 findings such as patient utilization, disease data, or service coverage, medicine stock out…………………	1.Yes, observed	2. No	Disc_DHIMS2
2.10.1 c. Have they made any decisions based on the above discussions?	1.Yes, observed	2. No	Dec_Disc
2.10.1 d. Has any follow-up action taken place regarding the decisions made during the previous meetings?...	1.Yes, observed	2. No	Fol_Dec
2.10.1 e. Are there any DHIMS 2 related issues or problems that were referred to the district or regional level for actions?...........................	1.Yes, observed	2. No	DHIMS2_Refer
2.11. *Observed* facility received annual/monthly planned targets based on DHIMS2 information? …………………………………………………….	1.Yes	2. No	Obs_Plan
2.12. Do facility records for the last three months show that district/senior Management issued directives concerning the use of DHIMS 2 information……	1.Yes	2. No	Iss_Direct
2.13. Did the facility receive a district or national DHIMS 2 office newsletter or report in last three months giving examples of use of information……………………	1.Yes	2. No	Fac_Rep
2.14. Does documentation exist showing the use of DHIMS 2 information for advocacy purposes?	1.Yes	2. No	Doc_Use
2.15. Did the person in charge of the facility participate in meetings at district level to discuss DHIMS 2 performance for the last three months?	1.Yes	2. No	Char_Part

2.16. Please give examples of how the facility uses DHIMS 2 information for health system management………	1. Yes (details follows)	2. No examples	Exam_Uses

2.17. How many times did the district supervisor visit your facility during the last three months? (check the answer)………………….

a. 0	b. 1	c. 2	d. 3	e. >3	Times_Super

If 0, go to 2.17.1c………………………………….

2.17.1a. Did the district supervisor discuss performance of health facilities based on DHIMS2 information when he/she visited your facility?	1.Yes	2. No	Super_Faci
2.17.1b. Did the supervisor help you make a decision based on information from the DHIMS 2?	1.Yes	2. No	Super_dec
2.17.1c. Did the supervisor send a report/feedback/note on the last two supervisory visits?	1.Yes	2. No	Super_Rep

3. Form checked and certified by?			FW_FS

END OF FORM. CHECK YOUR FORM AND THANK THE RESPONDENT
