# Supplementary material for: Utilization of the national cluster of district health information system for health service decision-making at the district, sub-district and community levels in selected districts of the Brong Ahafo region in Ghana
Source: BMC Health Serv Res. 2020 Jun 6;20:514. doi: 10.1186/s12913-020-05349-5 (PMC7275484; doi:10.1186/s12913-020-05349-5)
Supplement: Supplementary file 3 — Additional file 3. Behavioral Assessment Form. The behavioral assessment form is a quantitative interview guide used to collect data from all selected participants during the study. [file 12913_2020_5349_MOESM3_ESM.pdf]

KINTAMPO HEALTH RESEARCH CENTRE<br><br>BEHAVIOURAL ASSESSMENT FORM<br><br>DHIMS 2 EVALUATION STUDY 06/07/2017	FORM NO	FORMNO

1. BASIC INFORMATION:

1.1. Village code and name? ………………				V_code
1.2. Name of facility				Fac_name
1.3. Date of visit……………………				Day_visit

1.4. Type of facility?

1. Hospital	2.Health Centre	3. Private Clinic	4. CHPS	Type_hf
5. DHMT facility (eg Kintampo RCH)		6. Other, specify….		

1.5. ENTER FACILITY CODE: ………………………………………………… Hf_code

1.6. Respondent Name ______________________________ R_name

1.7. Sex of respondent ………	1. Male	2.Female	R_sex

1.8. Religion?	1. Christian	2.Muslim	3. Traditional	4. Other, specify..	R_religion

1.9. Marital status?	1.Married	2. Never married/single	3. Divorced	4. Co-habiting	R_maristatus

1.10. Highest educational level? ...	1. Certificate	2. Diploma	3. Graduate degree/above	R_edulevel

1.11. What is your profession?

1. Med. doctor	2.Med. assistant	3. RN/PHN	4. Lab tech	5. Pharmacist	R_profession

6. Biostatistician	6. Nutritionist	7. CHO	8. IT personnel	9. Other specify………

1.12. What is your job title?

1. Director	2.Administrator	3. Matron	4. In-charge	R_jobtitle
5. Head of department	6. Program/Project Coordinator	7. Other, specify		

1.13. How long have you been working in this facility? ....

Year			Years_work
Month			Months_work
Weeks			Weeks_work
Days			Days_work

1.14. How long have you been working in your current capacity? ....

Year			Cap_years
Month			Cap_months
Weeks			Cap_weeks
Days			Cap_days

2. ORGANIZATIONAL AND BEHAVIOURAL ASSESSMENT TOOL

(TO BE FILLED BY STAFF AND MANAGEMENT AT ALL LEVELS)

We would like to know your opinion about how strongly you agree with the following activities. There are no right or wrong answers, but only expression of your opinion on a scale. The scale is about assessing the intensity of your belief and ranges from strongly disagree (1) to strongly agree (7).

This information will remain confidential and would not be shared with anyone, except presented as an aggregated data report. Please be frank and choose your answer honestly.

Strongly disagree<br>1	Disagree<br>2	Somewhat Disagree<br>3	Neither Disagree nor Agree<br>4	Somewhat Agree<br>5	Agree<br>6	Strongly Agree<br>7

2.1To what extent, do you agree with the following on a scale of 1-7?

2.1.1 **In health department, decisions are based on**

	Strongly Disagree	Somewhat Disagree	Disagree	Neither Disagree nor Agree	Agree	Somewhat Agree	Strongly Agree	
2.1.1a Personal liking	1	2	3	4	5	6	7	Pers_Lik
2.1.1b Superiors' directives	1	2	3	4	5	6	7	Supe_Dir
2.1.1c Evidence/facts	1	2	3	4	5	6	7	Evi_Fac
2.1d Political interference	1	2	3	4	5	6	7	Pol_Int
2.1.1e. Comparing data with strategic health objectives	1	2	3	4	5	6	7	Com_Stra
2.1.1f. Health needs	1	2	3	4	5	6	7	Hea_Needs
2.1.1g. Considering costs	1	2	3	4	5	6	7	Cons_Cost

2.1.2 In health department, superiors

	Strongly Disagree	Somewhat Disagree	Disagree	Neither Disagree nor agree	Agree	Somewhat Agree	Strongly Agree	
2.1.2a Seek feedback from concerned persons	1	2	3	4	5	6	7	Feed_Con
2.1.2a Emphasize data quality in monthly reports	1	2	3	4	5	6	7	Emph_Qua
2.1.2a Seek feedback from concerned community	1	2	3	4	5	6	7	Feed_Com
2.1.2a Use DHIMS 2 data for setting targets and monitoring	1	2	3	4	5	6	7	Dhims_Tar
2.1.2a Check data quality at the facility and higher level regularly	1	2	3	4	5	6	7	Che_Reg
2.1.2a Provide regular feedback to their staff through regular report based on evidence	1	2	3	4	5	6	7	Staff_Evi
2.1.2a Report on data accuracy regularly	1	2	3	4	5	6	7	Rep_Acc

2.1.3 In health department, staff

2.1.3b. Document their activities and keep records	1	2	3	4	5	6	7	Doc_Rec
2.1.3c. Feel committed in improving health status of the target population	1	2	3	4	5	6	7	Comm_Tar
2.1.3d. Set appropriate and doable target of their performance	1	2	3	4	5	6	7	App_Perf
2.1.3e. Feel guilty for not accomplishing the set target/performance	1	2	3	4	5	6	7	Guil_Acc
2.1.3f. Are rewarded for good work	1	2	3	4	5	6	7	Rew_Good

	Strongly Disagree	Somewhat Disagree	Disagree	Neither Disagree nor agree	Agree	Somewhat Agree	Strongly Agree	
**2.1.4 In health department, staff**								
2.1.4a. Use DHIMS 2 data for day to day management of the facility and district	1	2	3	4	5	6	7	Day_Man
2.1.4b. Display data for monitoring their set target	1	2	3	4	5	6	7	Dis_Mon
2.1.4c. Can gather data to find the root cause(s) of the problem	1	2	3	4	5	6	7	Root_Pro
2.1.4d. Can develop appropriate criteria for selecting interventions for a given problem a	1	2	3	4	5	6	7	Dev_Crit
2.1.4e. Can develop appropriate outcomes for a particular intervention	1	2	3	4	5	6	7	Dev_Out
2.1.4f. Can evaluate whether the targets or outcomes have been achieved	1	2	3	4	5	6	7	Eva_Ach
2.1.4g. Are empowered to make decisions	1	2	3	4	5	6	7	Emp_Dec
2.1.4h. Able to say no to superiors and colleagues for demands/decisions not supported by evidence	1	2	3	4	5	6	7	Not_Evi
2.1.4i. Are made accountable for poor performance	1	2	3	4	5	6	7	Acc_Poor
2.1.4j. Use DHIMS 2 data for community education and mobilization	1	2	3	4	5	6	7	Com_Mob

2.1.5 Personal

2.1.5a. Collecting information which is not used for decision making discourages me	1	2	3	4	5	6	7	Col_Dis
2.1.5b. Collecting information makes me feel bored	1	2	3	4	5	6	7	Col_Bored
2.1.5c Collecting information is meaningful for me	1	2	3	4	5	6	7	Col_Mean
2.1.5d Collecting information gives me the feeling that data is needed for monitoring facility performance	1	2	3	4	5	6	7	Col_Mon
2.1.5e Collecting information gives me the feeling that it is forced on me	1	2	3	4	5	6	7	Col_Force
2.1.5f Collecting information is appreciated by co-workers and superiors	1	2	3	4	5	6	7	Col_App

This part of the questionnaire is about your perceived confidence in performing tasks related to DHIMS2. High confidence indicates that person could perform the task, while low confidence means room for improvement or training. Please be frank and rate your confidence honestly.

Please rate your confidence in percentages that you can accomplish the DHIMS 2 activities.

3.1 Rate your confidence for each situation with a percentage from the following scale

0 10 20 30 40 50 60 70 80 90 100

3.1a. I can check data accuracy	0	10	20	30	40	50	60	70	80	90	100
3.1b. I can calculate percentages/rates correctly	0	10	20	30	40	50	60	70	80	90	100
3.1c. I can plot data by months or years	0	10	20	30	40	50	60	70	80	90	100
3.1d. I can compute trend from bar charts	0	10	20	30	40	50	60	70	80	90	100
3.1e. I can explain findings & their implications	0	10	20	30	40	50	60	70	80	90	100
3.1f. I can use data for identifying gaps and setting targets	0	10	20	30	40	50	60	70	80	90	100
3.1g. I can use data for making various types of decisions and providing feedback	0	10	20	30	40	50	60	70	80	90	100

3.3 Form checked and certified by [ ][ ] FW_FS

END OF FORM. CHECK YOUR FORM AND THANK THE RESPONDENT
