# Supplementary material for: Utilization of the national cluster of district health information system for health service decision-making at the district, sub-district and community levels in selected districts of the Brong Ahafo region in Ghana
Source: BMC Health Serv Res. 2020 Jun 6;20:514. doi: 10.1186/s12913-020-05349-5 (PMC7275484; doi:10.1186/s12913-020-05349-5)
Supplement: Supplementary file 4 — Additional file 4. Desk Review Guide. The desk review guide is an observational interview guide that was used to guide the review of documented evidence all three levels of the district health system during the study. [file 12913_2020_5349_MOESM4_ESM.pdf]

KINTAMPO HEALTH RESEARCH CENTRE<br><br>DESK REVIEW GUIDE<br><br>DHIMS 2 EVALUATION STUDY 06/07/2017	FORM NO.	**FORM NO.**

No.	Item	Response			Code
1.	Name of the Facility				Nam_Fac
2.	Name of the District				Nam_Dist
3.	Name of the Assessor				Nam_Ass
4.	Date of Assessment				Dat_Ass
5.	Presence of management structure for dealing with DHIMS 2 related strategic and policy decisions at district and higher levels	1.Yes	2.No		Pres_Manag
6.	Presence of an updated (last year) district health management organizational chart, showing functions related to DHIMS 2/health information	1.Yes	2.No		Pres_Chart
7.	Presence of distribution list and documentation of DHIMS 2 past monthly/quarterly report distribution at district or higher level	1.Yes	2.No		Pres_Dist
8.	Presence of DHIMS 2 situation analysis report? ……………………………………………………	1.Yes	2.No		Pres_Ana
9.	Presence of DHIMS 2 targets at facility and higher level? …………………………………….	1.Yes	2.No		Pres_Targ
10.	Presence of a copy of DHIMS 2 standards at district or higher levels? …………………….	1.Yes	2.No		Pres_Dtand
11.	Presence of a copy of DHIMS 2 standards at the facility? …………………………………….	1.Yes	2.No		Pres_Fstand
12.	Presence of performance improvement tools (flow chart, control chart etc.) at the facility?..	1.Yes	2.No		Pres_Improv
13.	Does facility/district have a DHIMS 2 training manual?	1.Yes	2.No		Fac_Manual
14.	Presence of mechanisms for on-job DHIMS 2 training (see documentation)? ………	1.Yes	2.No		Pres_Onjob
15.	Presence of schedule for planned training	1. Yes, for one year	2. Yes, 2 years or more	2.No	Pres_ Sched
16.	Presence of DHIMS 2 supervisory checklist	1.Yes	2.No		Pres_Super
17.	Presence of schedule for DHIMS 2 supervisory visit	1.Yes	2.No		Pres_Visit
18.	Presence of supervisory reports	1.Yes	2.No		Pres_Report
19.	Presence of DHIMS 2 related expense register	1.Yes	2.No		Pres_Expe
