# Supplementary material for: Utilization of the national cluster of district health information system for health service decision-making at the district, sub-district and community levels in selected districts of the Brong Ahafo region in Ghana
Source: BMC Health Serv Res. 2020 Jun 6;20:514. doi: 10.1186/s12913-020-05349-5 (PMC7275484; doi:10.1186/s12913-020-05349-5)
Supplement: Supplementary file 5 — Additional file 5. In-depth Interview Guide. The in-depth interview guide is a qualitative interview guide that was used to collect qualitative data for the purpose of the study. This file is in PDF format [file 12913_2020_5349_MOESM5_ESM.pdf]

KINTAMPO HEALTH RESEARCH CENTRE<br><br>IN-DEPTH INTERVIEW GUIDE<br><br>DHIMS 2 EVALUATION STUDY 06/07/2017	FORM NO	FORMNO

1. Briefly, describe what you know of DHIMS 2 and how it should routinely be utilised?

2. How often do you submit reports to DHIMS 2?

2.1. *If not so often, why is health information routinely not gathered?*

3. Does the health facility go through recommended procedures to process health-related data for submission to DHIMS 2?

3.1. *If yes, what procedures does it go through to process data?*

3.2. *If No, why doesn't the health facility go through the recommended process to process health related data?*

4. Does the facility's habits promote decision-making based on data from DHIMS 2?

4.1. *How*?

5. Does the facility have set of demands that prevents effective utilisation of DHIMS 2 data?

5.1. *If yes, what are those demands and how do they prevent effective utilization of DHIMS2 data?*

6. Are there process-related challenges hindering routine utilisation of DHIM 2 data?

6.1. *If yes, what are they and how do they hindering routine utilisation of DHIM 2 data?*

7. In your own view, which technical-related factors influence the routine utilisation of information from DHIMS 2 data in health facilities?

8. How can the technical factors be eliminated such that DHIMS 2 information is utilized?

9. In your own view, which behavioral related factors influence the routine utilization of health

information in health facilities?

10. How can the behavioral factors be eliminated such that routine health information is utilized?

11. In your own view, which organizational related factors influence the routine utilization of health information in health facilities?

12. How can the organizational factors be eliminated such that routine health information is utilized?

13. In your view, what can health facilities do to routinely utilize information from DHIMS 2?

14. Please describe examples of how the district office uses DHIMS 2 information for health system management.
